# Physicochemical Evaluation of Lyophilized Formulation of *p*-SCN-Bn-DOTA- and *p*-SCN-Bn-DTPA-rituximab for NHL Radio Immunotherapy

**Published:** 2016

**Authors:** Darinka Gjorgieva Ackova, Katarina Smilkov, Emilija Janevik-Ivanovska

**Affiliations:** *Faculty of Medical Sciences, Goce Delcev University-Štip, Republic of Macedonia.*

**Keywords:** Rituximab, Lyophilized formulation, *p*-SCN-Bn-DOTA, *p*-SCN-Bn-DTPA

## Abstract

Radioimmunotherapy (RIT) of Non-Hodgkin’s lymphoma (NHL) is said to be more advantageous compared to unlabelled therapeutic antibodies. To this date, radiolabelled murine anti-CD20 mAbs, Zevalin^®^ and Bexxar^®^ have been approved for imaging and therapy. A preparation containing rituximab, chimeric mAb radio immunoconjugate suitable for Lu-177 labeling, could provide better imaging and therapeutic profile at the same time. This study was conducted to evaluate prepared lyophilized formulations of two rituximab immune conjugates, intended for immediate Lu-177 labeling, for imaging and therapy.

The characterization of the conjugates and demonstration of the integrity of the protein and purity after conjugation and lyophilization was performed by SDS-PAGE, FT-IR and MALDI-TOF-MS. The results showed preserved antibody structure and average of 6.1 *p*-SCN-Bn-DOTA and 8.8 *p*-SCN-Bn-DTPA groups per antibody molecule which is suitable for successful labeling. These results support the possibility of developing a “ready-to-label” rituximab immune conjugates for NHL imaging/therapy.

## Introduction

Non-Hodgkin’s lymphoma (NHL) is a form of blood cancer with origin in lymphatic system. 

More than 90% of B-cell lymphoma cells express CD20 receptor, but it is not expressed on cells in stem and progenitor cell pools. CD20 has proven to be an excellent target for the treatment of B-cell lymphoma (1) and investigations for treatment of NHL have been based on the development of antibody against CD20 antigens (2). Results of these investigations have led to drugs such as rituximab, a chimeric antibody for immunotherapy of CD20-positive low-grade NHL, and ibritumomab and tositumomab, both murine antibodies, for the treatment of follicular lymphoma (3-5). Anti-CD20 monoclonal antibodies (mAbs) labeled with ^111^In (^111^In-ibritumomab) for imaging, ^90^Y (^90^Y-ibritumomab, Zevalin) for therapy and ^131^I (^131^I-tositumomab, Bexxar) for imaging and therapy have been approved for use in patients with NHL (5-8). Radioimmunotherapy (RIT), is said to be more advantageous compared to unlabelled therapeutic antibodies, but RIT with murine antibodies often has limitations like development of human anti-mouse antibodies (HAMA) (9). To overcome these limitations, rituximab radioimmune conjugates are under investigation for RIT. Various radionuclides, among them ^90^Y (10, 11), ^111^In (10-12), ^64^Cu (13), ^153^Sm (14), ^177^Lu (9, 10, 15), attached to antibody through different chelating agents, have been described or are under development. A number of chelating agents have been investigated for labeling proteins and peptides with radiometals, with various derivatives of the acyclic agent diethylene triamine pentaacetic acid (DTPA) and the macrocyclic agent 1,4,7,10-tetraazacyclododecane-1,4,7,10-tetraacetic acid (DOTA) being the most widely investigated (16).

Therapeutic monoclonal antibodies are very complex molecular structures that are joined together with weak, non-covalent or strong covalent, disulphide bonds, and their integrity is of exquisite importance for the physico-chemical stability and immunological potential. Proteins intended for therapy are often formulated in aqueous solution to allow ease of use, but it is known that aqueous environment can accelerate many degradation processes (17, 18). Although instabilities can be found in solid state also, the common approach of stabilization of therapeutic proteins is lyophilization. The lyophilization process is a trusted aseptic process operation meeting the product sterility assurance requirement without the stress of terminal sterilization (19). 

This work focuses on examination of ready-to-label lyophilized rituximab immune conjugates in order to increase the stability. In the search for a radioimmunoconjugate of higher efficiency and lower toxicity, obtained preparations of two bifunctional chelating agents (BFCA), *p*-SCN-Bn-DOTA and *p*-SCN-Bn-DTPA where compared. While preparations of these or other derivatives of DOTA and DTPA radio immune conjugates has been reported, chemical characteristics, stability and biodistribution of the prepared radioimmunoconjugates have not been explained in details. The main goal of this investigation was chemical characterization of the obtained immune complexes, analysis of identity, purity and post-lyophilization modifications.

## Experimental

*Materials and Methods*

*Materials*

*p*-SCN-Bn-DOTA [2-(4-isothiocyanatobenzyl)-1,4,7,10-tetraazacyclododecane-tetraacetic acid] and p-SCN-Bn-DTPA [2-(4-isothiocyanatobenzyl)-diethylenetriaminepentaacetic acid] with 94% purity were obtained from Macrocyclics Inc. (NJ, USA). Rituximab was purified from a commercial pharmaceutical sample (Mabthera^®^), purchased from Roche Co, CA, USA, using ultrafiltration (Ultracel^® ^- 30K, Millipore, Ireland) for concentration and buffer exchange to sterile 0.1 M PBS, pH 8.0. 

Matrix-Assisted Laser Desorption Ionization time-of-flight (MALDI-TOF) mass spectrometry was performed with Voyager-De MALDI-TOF (Applied Biosystems). Sodium dodecyl sulphate-polyacrylamide gel electrophoresis (SDS-PAGE) was performed on the mini-gel system (GE Healthcare/Amersham Biosciences). Fourier Transform Infrared (FT-IR) spectroscopy was applied for determination of protein secondary structure. FT-IR-Spectrometer, PARAGON 1000 (Perkin Elmer) was used for recording IR spectra of investigated compounds. 

*Conjugation of Rituximab to p-SCN-Bn-DOTA and p-SCN-Bn-DTPA*

BFCA’s were dissolved in 0.1 M PBS (pH 8.0) to final concentration of 10 mg/mL. Calculated amounts of BFCA required to give a 20-fold molar excess over the amount of Rituximab (10 mg/mL) were added to the purified monoclonal antibody in 0.1 M PBS (pH 8.0). The mixture was incubated overnight, at 4 °C with gentle shaking. Purification of the conjugates was made with ultrafiltration (Ultracel^® ^- 30K, Millipore, Ireland), by washing the immune conjugates with 0.05 M ammonium acetate, pH 7.0, until the absorbance in the ultrafiltrate set at 280 nm was nearly zero (meaning that there is no unbound chelating agent in the immune conjugate solution).

*Lyophilization Process*

The lyophilization was performed using Labconco Free Zone Stoppering Tray Dryer, (USA) using protocol described by Park *et al.* in 2013 (20), modified to our experience. Briefly, the liquid immunoconjugates were filled in 10 mL type I glass tubing vials using a fill volume of 1 mL loaded in the freeze-dryer. The temperature was decreased to -40 °C at 0.40 °C/min and held for 3 h, increased to -15 °C, to allow complete crystallization, thus completing the freezing step in 10 h. The primary drying was performed at temperature of -10 °C and the secondary drying at shelf temperature 25 °C. Upon finishing the process, the vials were stoppered and kept at 4 °C until analysis.

*Protein Integrity Test Using SDS-PAGE*

SDS-PAGE was performed according to Laemmli protocol (21). About 5 µL of sample was mixed with 10 µL of sample buffer. The samples were boiled for 5 min at 95 °C. Approximately 5 µL of each preparation was applied per lane in 12% bisacrylamide under reducing conditions. Coomassie staining (Coomassie Brilliant Blue R-250, Sigma) was performed according to the manufacturer’s instructions. All chemicals used were reagent and HPLC grade. As molecular marker Low molecular weight marker (Amersham GE Healthcare) was used.

*Protein Characterisation by MALDI-TOF MS*

Both characterization of the conjugates and determination of the average number of BFCA attached to each antibody molecule is performed by MALDI-TOF mass spectrometry. A representative procedure can be outlined as follows: A volume (10 µL) of the solution of the conjugated BFCA’s was diluted (1:10) with a matrix solution of 3,5-dimethoxy-4-hydroxycinnamic acid [10 mg/mL dissolved in a mixture of acetonitrile (50%)/TFA (1%), Sigma] to a concentration of about 10 pmol/μL. An aliquot (1-2 μL) of the final solution was applied to the sample target prior to insertion into the high vacuum chamber of a mass spectrometer. Operational conditions for the MALDI-TOF apparatus were set as follows: mode of operation, linear; polarity, positive; acceleration voltage, 20000 V; delayed extraction time, 100 nsec; aquisition mass range, 140000-170000 Da.

*FT-IR Spectroscopy*

The lyophilized samples were used for FT-IR study. The spectral range was 2000–500 cm^-1^ and the samples were scanned with ATR (Attenuated Total Reflectance) technique three times to minimize the influence of spotting variance. Spectra were acquired at a resolution of 4 cm^-1 ^and 128 spectra were co-added to improve the signal-to-noise ratio. After spectral acquisition, data were exported and analyzed using the Grams_32 software.

**Figure 1:**
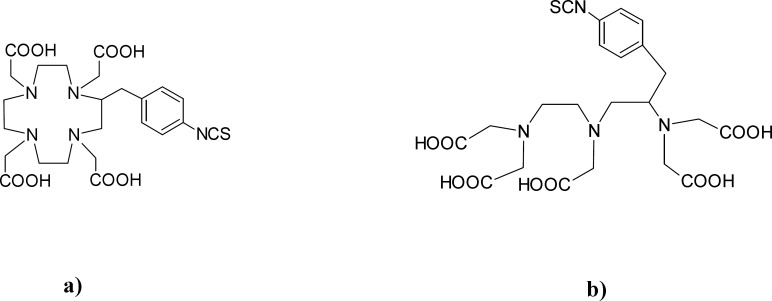
Structure of used BFCAs: a)* p*-SCN-Bn-DOTA, b)* p*-SCN-Bn-DTPA

**Figure 2:**
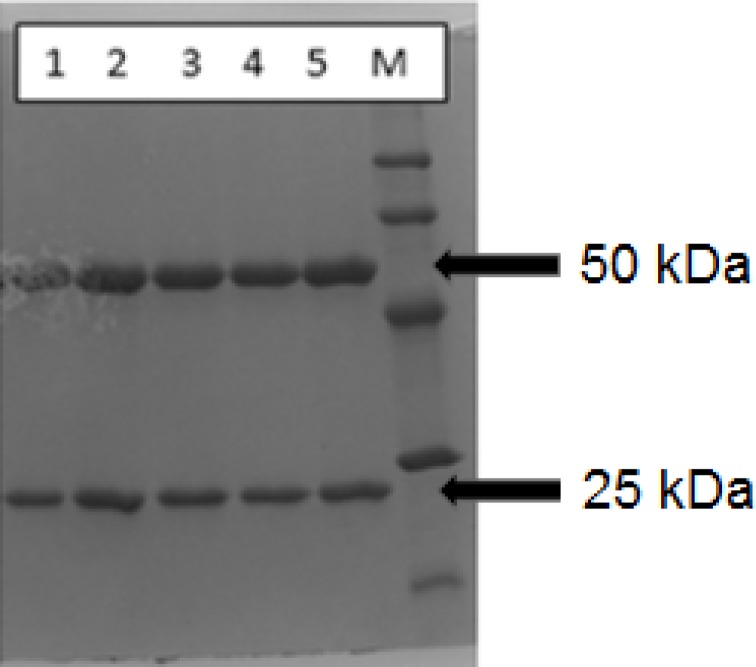
Reducing SDS-PAGE lane patterns for rituximab (1) (1 mg/mL), DOTA-rituximab conjugate, before lyophilization (2), DTPA-rituximab conjugate, before lyophilization (3), DOTA-rituximab conjugate, after lyophilization (4) and DTPA-rituximab conjugate, after lyophilization (5); M is molecular marker

**Figure 3:**
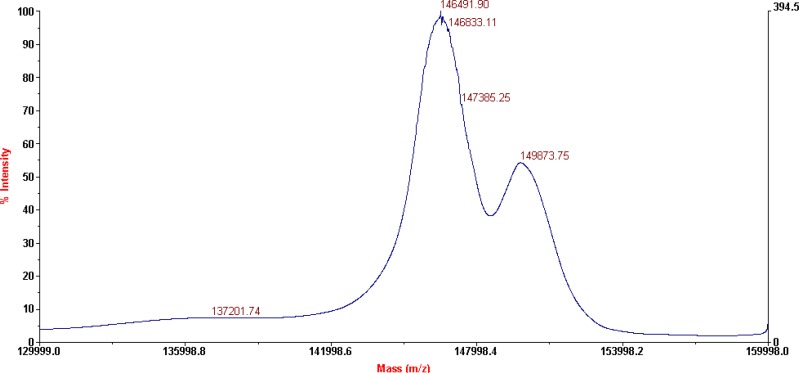
MALDI-TOF results for DOTA-rituximab conjugate

**Figure 4:**
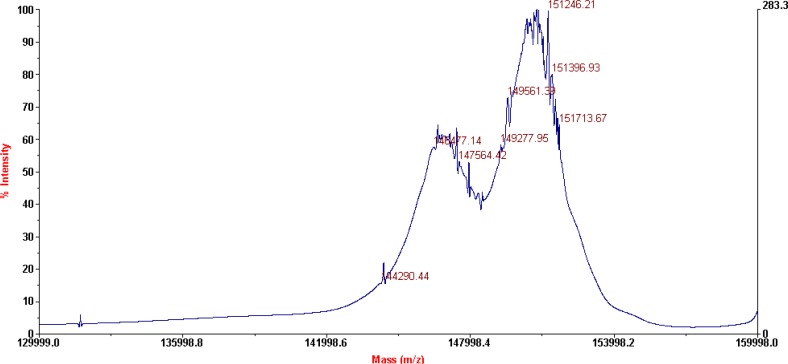
MALDI-TOF results for DTPA-rituximab conjugate

**Figure 5:**
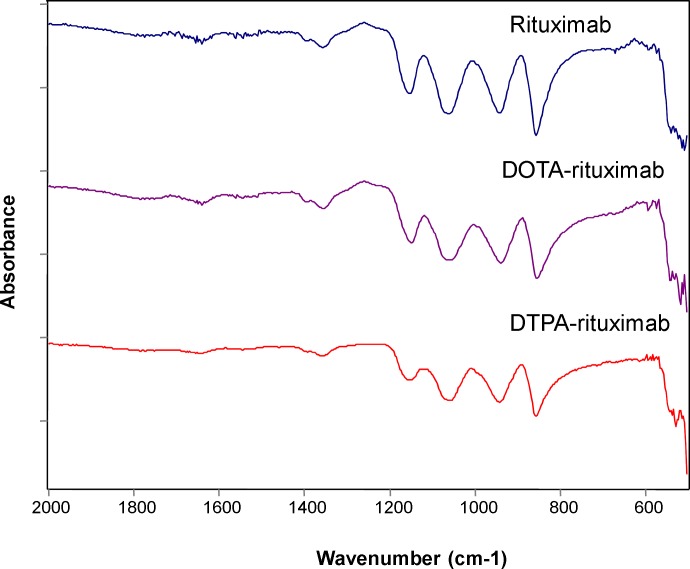
IR spectra of rituximab, DOTA-rituximab and DTPA-rituximab (after lyophilization

## Results and Discussion

The preparation of protein therapeutics as lyophilized (freeze-dried) products is often essential to obtain the mandatory stability during shipping and long-term storage.

Because of its protein nature, rituximab may go through a variety of chemical and physical degradation processes (22). Chemical instability, concerns covalent bond modifications (asparagine deamidation, oxidation or disulfide bond rearrangement) (23), while physical instability mostly concerns low energy bonds (hydrogen bonds), and includes adsorption on to surfaces, unfolding and aggregation (24). 

As it is an important challenge to access the stability of monoclonal antibodies as a part from biotechnology-derived radiopharmaceuticals, we conducted a study to evaluate the physicochemical stability of two ready-to-label lyophilized rituximab immunoconjugates. Various protein characterization methods were used to determine changes in physicochemical properties of rituximab conjugates after lyophilization.

In our study, as chelator molecules, derivatives of DOTA and DTPA were employed, and their structures are shown in Figure 1.

In order to demonstrate the integrity of the protein and purity after conjugation and lyophilization, SDS-PAGE was performed using 12% bisacrylamide gel. The loaded samples were Rituximab (1 mg/mL, commercial sample), conjugated *p*-SCN-Bn-DOTA, *p*-SCN-Bn-DTPA in liquid form and reconstituted lyophilized formulation, both in concentration of 1 mg/mL Figure 2. shows the SDS-PAGE patterns for BFCA conjugates, compared to unconjugated rituximab as control sample.

All BFCA-rituximab conjugates (before and after lyophilization) were resolved in two distinct Mw species which migrated in two bands (upper band at ~50 kDa and lower band at ~25 kDa) confirming the migration behavior typical for IgG antibodies which are composed of two identical subunits each composed by two polypeptide chains: two heavy and two light chains, linked via disulfide bonds. The obtained fragments correspond to molecular masses of rituximab heavy and light chain given in theliterature (25). 

As it is shown in Figure 2. the reducing SDS-PAGE patterns for rituximab, and BFCA-rituximab immunoconjugates were with very similar intensity. The reducing SDS-PAGE results, compared to the result of commercially available rituximab sample, showed no clear indication for antibody degradation. Similar results were obtained for one month integrity test on stored BFCA-rituximab immunoconjugates (results not shown). 

One of the most important quality attributes of the immuneconjugates is the average number of chelator molecules that are conjugated because this deter­mines the drug distribution and the amount of “payload” that can be delivered to the tumor cell and can directly affect both safety and efficacy. 

The characterization of the conjugates and the determination of the average number of BFCA attached to each antibody molecule were performed by MALDI-TOF MS, as shown on Figure 3. and 4. respectively for p-SCN-Bn-DOTA-rituximab conjugate and p-SCN-Bn-DTPA-rituximab conjugate.

This technique is a rapid and sensitive analytical tool for peptide and protein characterization. Used in a variety of modes, MALDI-MS provides information such as the molecular weight of an intact protein, peptide mass mapping from a tryptic digest, and peptide sequencing. MALDI-TOF MS as a “soft” ionization technique is suitable for thermolabile, nonvolatile compounds, especially those of high molecular mass and is used successfully in biochemical and biotechnological areas for the analysis of therapeutic proteins, peptides, glycoproteins, complex carbohydrates and oligonucleotides. One of the first reports of mass spectroscopic characterization of immuno conjugates describes use of a UV MALDI-TOF instrument (26, 27), where mass spectra of intact antibodies conjugated through lysine residues or through antibody carbohydrates with chelating agents (DTPA, macro­cycle 12N4) or with drugs (calicheamicin, methotrexate, mito­xantrone) were compared with the corresponding unconjugated antibodies.

MALDI-TOF results for P-SCN-Bn-DOTA-rituximab conjugate, after lyophilization (shown on Figure 3), revealed the presence of two major peaks corresponding to a Mw of 146491 Da (unconjugated mAb), and 149873 Da (conjugated mAb) which corresponds to an average of 6.1 groups of* p*-SCN-Bn-DOTA per molecule of rituximab. No structural changes in terms of appearance of additional peaks were observed. 

MALDI-TOF results for p-SCN-Bn-DTPA-rituximab conjugate, after lyophilization (shown on Figure 4), revealed the presence of two major peaks also, corresponding to a Mw of 146477 Da (unconjugated mAb), and 151246 Da (conjugated mAb) which corresponds to an average of 8.8 groups of* p*-SCN-Bn-DTPA per molecule of rituximab.

According to literature data (9), 4.25 ± 1.04 DOTA-SCN molecules attached to each antibody molecule are found to be sufficient for prompt subsequent labelling with radioisotope. This is result for investigated molar ratio 1:50 (antibody:chelator). Our results of average of 6.1 groups of* p*-SCN-Bn-DOTA and 8.8 groups of* p*-SCN-Bn-DTPA per molecule of rituximab, pointed that this number can be increased using different molar ratios for conjugation, as 1:20 in this case. 

In another study, up to five DOTA molecules were conjugated to MORAb-003, with no apparent loss of immunoreactivity (28). Highly DOTA-substituted anti-tumor antibody leads to the formation of immunoconjugates with high specific activity and excellent in-vivo behavior which is a valuable option for radioimmunotherapy and potentially antibody-drug conjugates (29).

Infrared (IR) spectroscopy appears as valuable method for monitoring protein denaturation upon lyophilization (30), although other methods have also been used such as mass spectroscopy (31), and Raman spectroscopy (32). In this study, the secondary structure of the protein in dried state was monitored using FT-IR spectroscopy. The IR spectra of the investigated compounds were recorded in the region 500-2000 cm^-1^ and compared to the IR spectra recorded for unconjugated rituximab. The results are shown on Figure 5. 

Each type of secondary structure (i.e. α-helix, β-sheet, β-turn and disordered) gives rise to different C=O stretching band frequencies. Most structural information is obtained by analysis of the conformationally-sensitive amide I band, which is located between 1600 and 1700 cm^-1^ (33, 34). According to previous investigations (33), a strong amide II band is observed at 1540–1550 cm^-1^ and a weaker shoulder at 1510–1525 cm^-1^. Antibody molecules are predominantly made of *β*-sheet (47%), 7% of *α*-helices, and the remaining percentage, of turns and coils (35). For all samples, namely, rituximab (1619; 1636; 1687 cm^-1^), P-SCN-Bn-DOTA-rituximab (1638; 1656; 1678 cm^-1^), and DTPA-rituximab (1636; 1656; 1679 cm^-1^) in the amide I region, in the recorded IR spectra (Figure 5.), we detected predominantly bands characteristic for β-structure. 

These findings are in accordance with literature data (33, 34). No modification in the obtained IR spectra of conjugates was observed, and in correlation with stability as indicated by results obtained by SDS-PAGE, the results revealed maintenance of the antibody native structure. Based on these results, we can conclude that conjugation and lyophilization process did not affected structure properties and caused no post-lyophilization modifications justifying the use of these formulations in further investigations for subsequent radionuclide labeling. The assignment of immunoconjugates (monoclonal antibodies with preserved secondary β-sheet structure of rituximab) as well as detailed analysis of characteristic bands shifts in rituximab vibrational spectra (depending on the presence of different chelators) is in progress.

## Conclusions

Our results demonstrate that after lyophilization, diluted (1 mg/mL in saline) rituximab immunoconjugates remain stable. Indeed, no modification of its chemical, physical and structural characteristics and no aggregation were observed. Further experiments are needed in order to demonstrate their biological and pharmacological properties. These results indicate that the time frame for the practical use of rituximab immune conjugates can be safely extended using lyophilization, allowing, for example, safe and longer storage. Our results also support the possibility of preparing standardized batches of “ready-to-label” rituximab immuno conjugates, following good manufacturing procedures. This can be a good base for conducting further experiments with radiolabeled formulations in order to develop a new promising radiopharmaceutical for therapy of NHL.
